# Editorial: Women in microbiome in health and disease 2021

**DOI:** 10.3389/fcimb.2022.1054190

**Published:** 2022-10-11

**Authors:** Maria de Lourdes Pereira, Maayan Levy, Veeranoot Nissapatorn, Gislane Lelis Vilela de Oliveira

**Affiliations:** ^1^ Centre for Research in Ceramics and Composite Materials (CICECO) - Aveiro Institute of Materials & Department of Medical Sciences, University of Aveiro, Aveiro, Portugal; ^2^ Microbiology Department, Perelman School of Medicine, University of Pennsylvania, Philadelphia, PA, United States; ^3^ School of Allied Health Sciences and World Union for Herbal Drug Discovery [WUHeDD], Walailak University, Nakhon Si Thammarat, Thailand; ^4^ Institute of Biosciences, Humanities and Exact Sciences (IBILCE), São Paulo State University (UNESP), Sao Jose do Rio Preto, Brazil

**Keywords:** women in science, pregnancy, women health, microbiome, dysbiosis

Currently, less than 30% of researchers worldwide are women. Long-standing biases and gender stereotypes are discouraging girls and women from moving away from science-related fields, and STEM research in particular. Science and gender equality are, however, essential to ensure sustainable development, as highlighted by UNESCO and the United Nations. To change traditional mindsets, gender equality must be promoted, stereotypes defeated, and girls and women should be encouraged to pursue STEM careers.

This Research Topic of Frontiers in Cellular and Infection Microbiology celebrates the achievements of women in science and highlights the diversity of research performed across the entire breadth of the microbiome in health and disease research, and presents advances in theory, experimental, and methodology with applications to compelling problems. A total of 14 articles authored by women were published and 97 researchers participated in this Research Topic. **Figure 1** summarizes the main findings of the articles in relation to associations between microbiomes and women health.

**Figure 1:**
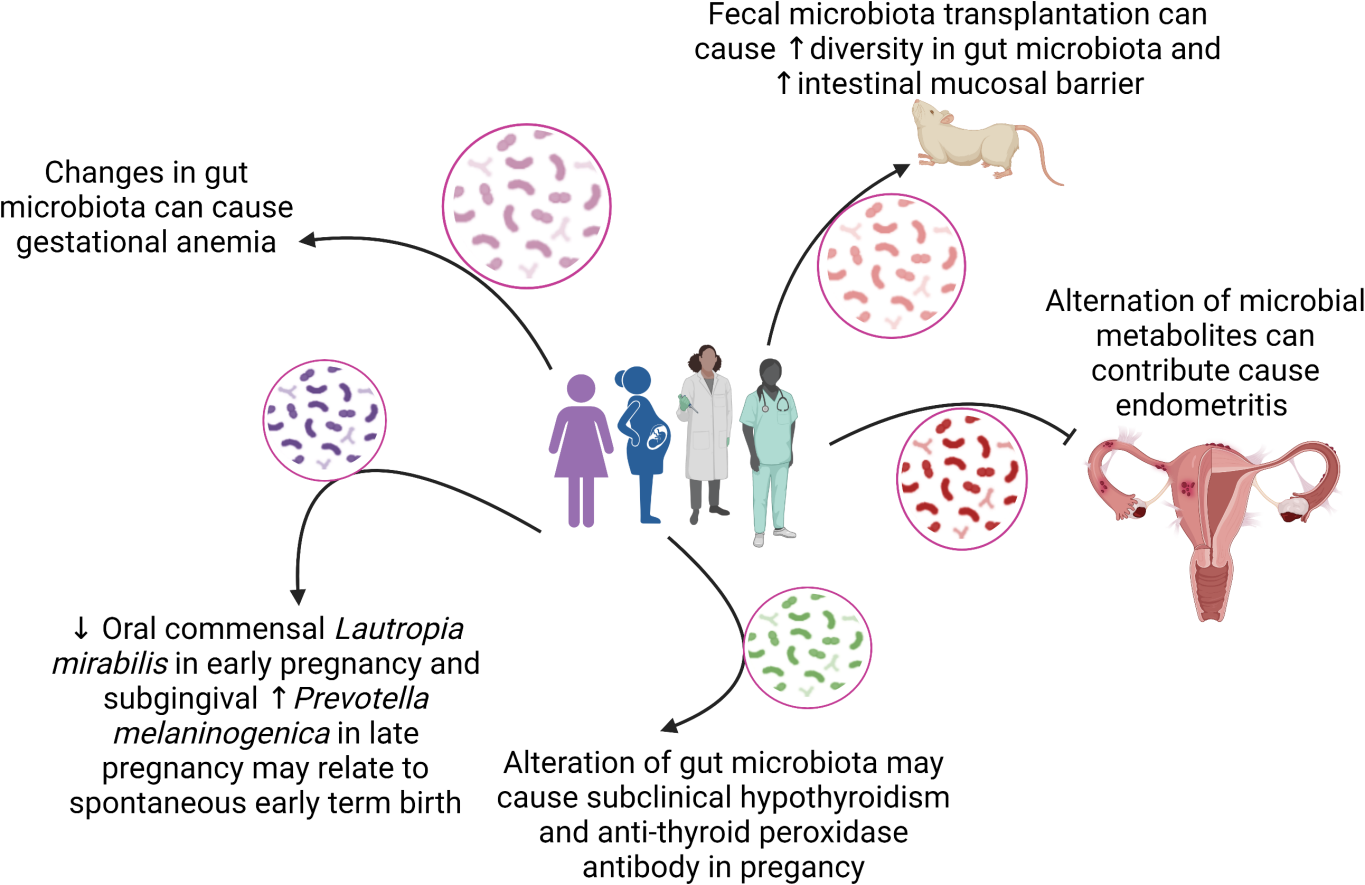
The association between microbiomes and women health.

Emerging findings from animal models and human pregnancy studies have shown that different factors affect maternal and fetal complications, including intrauterine or extrauterine infections, oral or intestinal dysbiosis, and dysregulated immune responses (Romero et al., 2014; MacIntyre et al., 2015; Piler et al., 2017; Fettweis et al., 2019; Serrano et al., 2019; Jehan et al., 2020; Pansieri et al., 2020; Kumar et al., 2021). Two mechanisms have been proposed to explain how gut microbes move into the uterus: 1) gram-negative microbes induce mediators of inflammation, such as prostaglandins that facilitate the ascent of microbes through the vagina; 2) intestinal hyperpermeability during pregnancy promotes bacterial translocation to the uterus or placenta (Edwards et al., 2017). Kumar et al. revised and discussed how pathogens can cross the placental barrier and promote undesirable outcomes in the pregnancy, in the childbirth and the newborn. Also, they suggest that vaginal dysbiosis can induce an abnormal immune response in pregnant women and function as predictor marker for adverse outcomes from congenital infections.

Regarding dysbiosis, some studies have suggested the connection between alterations in the diversity and function of the oral, gut and vaginal microbiota and pregnancy complications (Borgo et al., 2014; Cobb et al., 2017; Fujiwara et al., 2017; Goltsman et al., 2018). For example, oral dysbiosis with decreased *Lactobacillus* and increased *Porphyromonas gingivalis, Neisseria*, and *Treponema* may contribute to preeclampsia, miscarriage, preterm labor and low birth weight (Cobb et al., 2017; Fujiwara et al., 2017; Goltsman et al., 2018; Jang et al., 2021). Interestingly, the presence of *Fusobacterium nucleatum* in the amniotic fluid of preterm labor pregnant women indicates oral microbes translocation to the placenta (Nuriel-Ohayon et al., 2019; Amir et al., 2020). The gut microbiota of pregnant women with or without complications differs significantly, with a greater abundance of opportunistic pathogens in the first group, impacting maternal metabolism and fetal development (Koren et al., 2012; DiGiulio et al., 2015; Edwards et al., 2017; Jiang et al., 2021).

The composition of the vaginal microbiota during pregnancy changes significantly and the decrease in *Lactobacillus* in this environment is associated with preterm birth (Aagaard et al., 2012; Di Simone et al., 2020; Marangoni et al., 2021). Zakaria et al. revised several physiological changes in the intestinal, oral and vaginal microbiomes during pregnancy, relating these alterations to mother and fetal health. In addition, researchers finalized by discussing the effect of probiotics to manage the microbiome during pregnancy. Similarly, Dreisbach et al. provided us an updated overview of the impact of obesity on maternal gut microbiota and metabolism thought animal model studies. Researchers also discussed reports on probiotic applications in these settings.

The gestational anemia (GA) is associated with adverse maternal and fetal outcomes, including preterm birth, low birth weight, neonatal and maternal mortality (Guignard et al., 2021; Shi et al., 2022). In a prospective study, Wei et al. evaluated the gut microbiota in GA (n = 156) and in healthy pregnant women (n = 402). Alpha and beta diversities were calculated and researchers showed significant differences between microbial communities in the third trimester, compared to the second trimester, in addition to the differential relative abundance of *Megamonas*, *Veillonella*, and *Haemophilus* when compared to healthy controls. A microbial co-abundance group network predicted upcoming anemia in healthy pregnant women with an area under the ROC curve of 0.7738 (95%CI: 0.7171, 0.8306), suggesting the possibility of identifying women at high-risk for the GA development, and the gut microbiota as a target for therapeutic modulation, through the use of functional foods and probiotics.

In a single-center prospective observational study, Wu et al. evaluated the fecal microbiota and metabolic functions in pregnant women with subclinical hypothyroidism, with (n = 75) or without (n = 90) positivity for anti-thyroid peroxidase antibodies (TPO). The group was also subdivided according to the levothyroxine (LT4) therapy (no treatment *vs*. low-dose LT4 *vs*. high-dose LT4). Researchers did not find significant differences in microbiota richness and evenness when comparing TPO positive and TPO negative women, however, they observe significant microbial diversity between TPO+ and TPO- in the high-dose LT4 group.

Using LEfSe analysis, authors detected a microbial signature related to the LT4 replacement, such as decreased species of *Ruminococcus* and *Bacteroides massiliensis* in low-and high-dose LT4 groups, respectively, as well as nineteen differential metabolic functions involved in lipid and amino acid metabolism discriminating TPO+ and TPO- pregnant women in the second trimester. The subclinical hypothyroidism in TPO+ pregnant women was normally associated with miscarriage, preterm birth, pre-eclampsia and gestational diabetes. Since the intestinal microbiota can affect the synthesis and functions of thyroid hormones (Knezevic et al., 2020; Fenneman et al., 2020; Bargiel et al., 2021), the study suggests microbiota modulation as a therapeutic option to treat TPO+ pregnant women.

In a very interesting longitudinal descriptive study, Yang et al. evaluated the subgingival microbiota in 59 pregnant women, from 8 to 14 weeks and from 24 to 30 weeks, correlating this data with gestational age and birth outcomes. No significant differences were observed in the richness, evenness and diversity of microbiota between 8 to 14 and 24 to 30 weeks of gestation; however, in the latter group, alpha and beta diversities were different between women who had early term births and those who had delivered at term.

Interestingly, the top twenty taxa represented in the subgingival microbiota of participants throughout pregnancy include bacteria involved in the progression to periodontal disease, including *Porphyromonas gingivalis*, *Tannerella forsythia*, *Prevotella*, and *Campylobacter* species. The oral dysbiosis is associated with periodontitis in pregnant women, and this disease is an important risk factor for preterm births (Jang et al., 2021; Saadaoui et al., 2021). In the present study, researchers identified that a decrease in *Lautropia mirabilis *at 8 to 14 weeks and an increase in *Prevotella melaninogenica *at 24 to 30 weeks of pregnancy were both associated with spontaneous early term birth and represent an important target for future studies.

The dietary habits during intrauterine life and after birth have an important impact on the health of the newborn´s microbiota and on the establishment of the adulthood microbiota later in life (Cadenas-Sanchez et al., 2017; Singh and Mittal, 2020). The gut microbiota colonization begins in the uterus and at birth, acquiring microbes from the mother and the environment. The main factors for the microbiota composition in early life are delivery type (normal or C-section), breastfeeding, time of introduction of solid foods, antibiotic use, and hygiene conditions (Martin et al., 2016; Le Doare et al., 2018). In a cross-sectional study in Brazil, Freitas et al. examined early-life data, body mass index (BMI), and collected fecal samples from 114 women with a mean age of 28 years and a mean BMI of 24.5 kg/m^2^. Beta diversity analysis of the microbiota showed two microbiota profiles, one driven by the *Blautia* genus (n = 56) and another driven by *Prevotella* (n = 58). The breastfeeding and adequate nutritional status were positively correlated with increased abundance of *Blautia, Anaerostipes*, and *Lachnoclostridium*. The exclusive breastfeeding (≥ 6 months) is associated with *Blautia*-driven profile of healthy women, showing the importance of early-life events and exclusive breastfeeding for infant gut colonization and health later in life.

The human milk oligosaccharides (HMO) are indigestible glycans that acts in the microbiota establishment and immunity maturation in the infant gut, and HMO are known to have bifidogenic effects (Bode, 2012; Wiciński et al., 2020). Kijner et al. investigated the mechanisms of HMO utilization by infant gut microbes by isolating *Bacteroides* strains from fecal samples, and testing them with the six most common HMO, 2′-fucosyllactose (2’-FL), difucosyllactose (DFL), 3′-sialyllactose (3’-SL), 6′-sialyllactose (6’-SL), lacto-N-tetraose (LNT), and lacto-N-neotetraose (LNnT). Differential RNA sequencing analysis showed that *Bacteroides dorei *presents an important glycoside hydrolase (GH) activity in break carbon bonds from HMO *in vitro*. Seventeen GH families were upregulated when cultivated with 2’-FL, 21 in DFL, 19 in 3’-SL, 23 in 6’-SL, 15 in LNT, and 18 in LNnT (log2 fold change > 1, *p* adj < 0.05), expanding our knowledge on the microorganisms involved in the HMO digestion, in addition to the already known *Bifidobacterium* species.

Regarding women’s health, in a multicenter, randomized, blinded clinical trial, Li et al. evaluated women with mixed aerobic vaginitis with bacterial vaginosis who received an effervescent suppository (n = 39) or clindamycin (n = 41). Women aged 20 to 55 years were randomized to receive either the suppository or clindamycin, vaginal swabs were collected at three time points (V1: -2~0 days; V2: 15-17 days; V3: 40 ± 3 days), and the DNA sequenced by Accurate 16S absolute quantification. Before treatment (V1), the pathogenic species *Gardnerella vaginalis, Atopobium vaginae, Sneathia amnii*, and *Prevotella bivia* were found in both groups.

After treatment, according to Shannon and Simpson indexes, the microbiota diversity significantly decreased in V2 in both groups (*p* < 0.001), and slightly increased in V3 in the suppository group. The absolute abundance of *Lactobacillus* increased in the suppository group compared to untreated patients, and the genera *Gardnerella, Prevotella*, and *Atopobium* were enriched in V3 in the clindamycin and suppository groups. Authors concluded that the effervescent suppository is effective to treat women with mixed aerobic vaginitis with bacterial vaginosis by restoring the vaginal microbiota.

Recent studies have investigated the relationship between the vaginal microbiota and cervical cancer (CC) to better understand the involvement of dysbiosis in the establishment, progression, or remission of the disease (Bray et al., 2018; Baezconde-Garbanati et al., 2019; Martínez-Rodríguez et al., 2021). In an observational cross-sectional study in Mexico, Manzanares-Leal et al. included 120 women aged between 21 to 71 years, 60 with advanced CC and 60 without the disease. Cervicovaginal swabs were collected to obtain culturable aerobic microbes, strains were evaluated by PCR-RFLP and identified by 16S sequencing. Researchers detected a specific microbiota associated with advanced stages of CC, including *Streptococcus urinalis, Escherichia coli, Bacillus safensis, Bacillus malikii, Corynebacterium jeikeium, Corynebacterium striatum*, and *Lactobacillus rhamnosus*. They concluded that there is no causal association between the aerobic vaginitis and cervicovaginal neoplasia, but proposed that vaginitis is fundamental for the progression of preneoplastic lesions to cancer (Vieira-Baptista et al., 2016; Plisko et al., 2021). Dong et al. contributed to a research article that aimed to assess the effects of acute endometritis on intestinal microbes and their metabolites. Endometritis is generally caused by bacterial infections, and accumulating evidence has shown that the occurrence of disease may be related to the gut microbiota (Plottel and Blaser, 2011; Borella et al., 2021). Moreover, the progression of diseases has previously been shown to change the composition and diversity of the intestinal microbiota and associated metabolites. The authors used a mouse model of endometritis that involves an intrauterine administration of lipopolysaccharide (LPS). Using 16S rRNA gene sequencing and liquid chromatogram-mass spectrometry, they found that the relative abundance of some members of the microbiome was changed and resulted in the reduction of beneficial microorganisms in the intestinal tract.

At the same time, acute endometritis increased the relative abundance of pathogenic bacteria, altered the concentration of intestinal metabolites, and affected biological oxidation, energy metabolism, and biosynthesis of primary bile acids. Thus, the findings of this study have the potential to provide new strategies for the diagnosis of acute endometritis.

Amyotrophic lateral sclerosis (ALS) is a heterogeneous neuromuscular disorder with progressive degeneration of the upper and lower motor neurons (Hardiman, 2021). A combination of genetic and environmental factors, as well as age‐related dysfunctions, are hypothesized to be involved in the ALS development (Masrori and Van Damme, 2020). Women are more affected by the disease with an estimation risk of 1:400 compared to 1:350 in men (Ryan et al., 2019; Masrori and Van Damme, 2020). Martin et al. contributed a review article that provides a comprehensive overview of the role that the microbiome may play in the ALS pathogenesis of. The authors explore existing evidence of gastrointestinal symptoms and microbial alterations in ALS pathogenesis from human and animal studies and discuss the possible therapeutic approaches to target specific diets, metabolites, and intestinal microbiome in ALS patients. They highlight innovative strategies for accurate diagnosis and better treatment for this challenging disease.

The last two articles discussed important aspects of fecal microbiota transplantation (FMT) in sepsis and bioinformatics tools for use in predictive models. Gai et al. reported that the FMT in sepsis model induced by cecal ligation and puncture is able to reestablish the gut microbiota diversity and decrease mortality by modulating the inflammatory response, restoring the epithelial barrier and function by upregulating the expression of tight junction proteins. Dahan et al. presented the EasyMap, an interactive online tool allowing for (1) running multiple multivariate linear regression models, with the same features and metadata; (2) visualizing the associations between microbial features and clinical metadata found in each model; and (3) comparing between the various models to identify critical metadata variables and select the optimal model. The EasyMap provides a side-by-side visualization of association results across the various models, each with additional metadata variables, enabling us to evaluate the impact of each metadata variable on the associated feature.

Collectively, the articles in this Research Topic demonstrated important aspects of the role played by the microbiomes from different sites (oral, gut and vagina) for women´s health, for a succeed pregnancy and fetal development, and for the prevention and treatment of diseases by using strategic approaches to modulate the microbiota, including nutritional interventions, probiotics and fecal microbiota transplantation. Furthermore, studies aimed at developing software for more accurate analyzes and prediction models are also necessary for the evolution of the microbiome field.

## Author contributions

GO and ML wrote the initial draft of the editorial, MP and VN revised the manuscript, and all authors approved the final version of the editorial.

## Acknowledgments

We would like to thank Dr. Alok K. Paul, University of Tasmania-Australia for assistance of an infographic using Biorender.com.

## Conflict of interest

The authors declare that the research was conducted in the absence of any commercial or financial relationships that could be construed as a potential conflict of interest.

## Publisher’s note

All claims expressed in this article are solely those of the authors and do not necessarily represent those of their affiliated organizations, or those of the publisher, the editors and the reviewers. Any product that may be evaluated in this article, or claim that may be made by its manufacturer, is not guaranteed or endorsed by the publisher.
